# 2-Amino­pyridin-1-ium triiodide

**DOI:** 10.1107/S1600536813015389

**Published:** 2013-06-08

**Authors:** Guido J. Reiss, Peer B. Leske

**Affiliations:** a Institut für Anorganische Chemie und Strukturchemie, Lehrstuhl II: Material- und Strukturforschung, Heinrich-Heine-Universität Düsseldorf, Universitätsstrasse 1, D-40225 Düsseldorf, Germany

## Abstract

The asymmetric unit of the title compound, C_5_H_7_N_2_^+.^I_3_^−^, consists of one 2-amino­pyridin-1-ium cation (*apy*H^+^) and one triiodide anion, both located in general postions. The *apy*H^+^ cation is planar within the experimental uncertainties. The short N—C distance [1.328 (5) Å] of the exocyclic NH_2_ group is typical for the imino-form of protonated 2-amino­pyridines. Consequently, the bond lengths within the six-membered ring vary significantly. The geometric parameters of the triiodide anion are in the typical range, with bond lengths of 2.8966 (3) and 2.9389 (3) Å and a bond angle of 176.02 (1)°. In the crystal, N—H ⋯ I hydrogen bonds connect adjacent ions into screwed chains along the *b*-axis direction. These chains are twisted pairwise into rectangular rods. The pyridinium moieties of neighbouring rods are arranged parallel to each other with a plane-to-plane distance of 3.423 (5) Å.

## Related literature

For the biological activity of amino­pyridines, see: Bolliger *et al.* (2011 ▶); Muñoz-Caro & Niño (2002 ▶). For amino­pyridinium salts with non-linear optical properties, see: Srinivasan & Priolkar (2013 ▶); Shkir *et al.* (2012 ▶); Periyasamy *et al.* (2007 ▶). For the spectroscopy of amino­pyridinium salts, see: Çırak *et al.* (2011 ▶). For bond-order calculations, see: Brown (2009 ▶). For the protonation and electronic structure of 2 amiopyridin-1-ium cations, see: Chapkanov (2010 ▶); Chai *et al.* (2009 ▶); Testa & Wild (1981 ▶). For the spectroscopy of polyiodides, see: Deplano *et al.* (1999) ▶. For pyridine–pyridine inter­actions, see: Ninković *et al.* (2012 ▶); Berl *et al.* (2000 ▶); Janiak (2000 ▶). For related poliodides, see: van Megen & Reiss (2012 ▶); Reiss & van Megen (2012*a* ▶,*b* ▶); Meyer *et al.* (2010 ▶); Reiss & Engel (2002 ▶, 2004 ▶). For the elemental analysis of polyiodides, see: Reiss & van Megen (2012*b* ▶); Egli (1969 ▶).
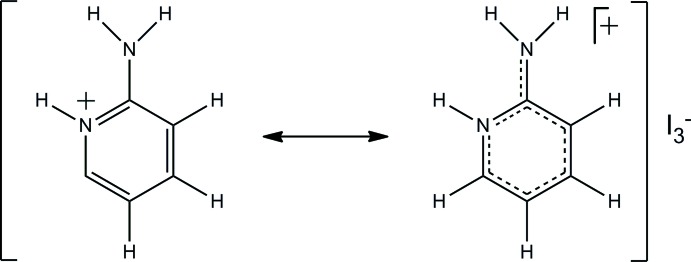


## Experimental

### 

#### Crystal data

C_5_H_7_N_2_^+^·I_3_^−^
*M* *_r_* = 475.83
Triclinic, 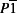

*a* = 8.0446 (4) Å
*b* = 8.9973 (5) Å
*c* = 9.1464 (4) Å
α = 117.805 (6)°
β = 90.939 (4)°
γ = 109.640 (5)°
*V* = 539.46 (6) Å^3^
*Z* = 2
Mo *K*α radiation
μ = 8.64 mm^−1^
*T* = 100 K
0.43 × 0.41 × 0.04 mm

#### Data collection

Oxford Diffraction Xcalibur Eos diffractometer
Absorption correction: analytical [*CrysAlis PRO* (Oxford Diffraction, 2009 ▶) based on expressions derived by Clark & Reid (1995 ▶)] *T*_min_ = 0.083, *T*_max_ = 0.698
5668 measured reflections
2186 independent reflections
2078 reflections with *I* > 2σ(*I*)
*R*_int_ = 0.021

#### Refinement

*R*[*F* ^2^ > 2σ(*F* ^2^)] = 0.018
*wR*(*F* ^2^) = 0.041
*S* = 1.01
2186 reflections
117 parameters
2 restraints
H atoms treated by a mixture of independent and constrained refinement
Δρ_max_ = 0.99 e Å^−3^
Δρ_min_ = −0.59 e Å^−3^

### 

Data collection: *CrysAlis PRO* (Oxford Diffraction, 2009 ▶); cell refinement: *CrysAlis PRO*; data reduction: *CrysAlis PRO*; program(s) used to solve structure: *SHELXS2013* (Sheldrick, 2008 ▶); program(s) used to refine structure: *SHELXL2013* (Sheldrick, 2008 ▶); molecular graphics: *DIAMOND* (Brandenburg, 2012 ▶); software used to prepare material for publication: *publCIF* (Westrip, 2010 ▶).

## Supplementary Material

Crystal structure: contains datablock(s) I, New_Global_Publ_Block. DOI: 10.1107/S1600536813015389/hg5319sup1.cif

Structure factors: contains datablock(s) I. DOI: 10.1107/S1600536813015389/hg5319Isup2.hkl

Additional supplementary materials:  crystallographic information; 3D view; checkCIF report

## Figures and Tables

**Table 1:** Hydrogen-bond geometry (Å, °)

*D*—H⋯*A*	*D*—H	H⋯*A*	*D*⋯*A*	*D*—H⋯*A*
N1—H11⋯I1	0.85 (1)	2.99 (3)	3.698 (3)	142 (4)
N1—H12⋯I3^i^	0.85 (1)	2.89 (2)	3.709 (3)	164 (4)
N2—H2⋯I1	0.83 (4)	2.97 (5)	3.702 (3)	147 (4)

Symmetry code: (i) 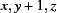.

**Fig. 1.**
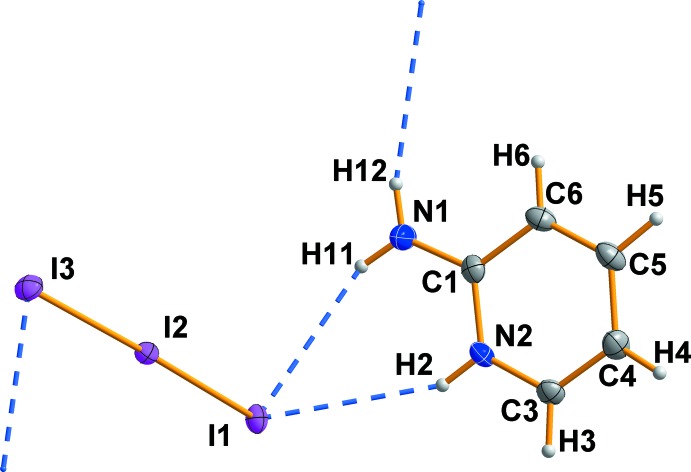
Showing the asymmetric unit of the title structure (Displacement ellipsoids are drawn at the 50% probability level).

**Fig. 2.**
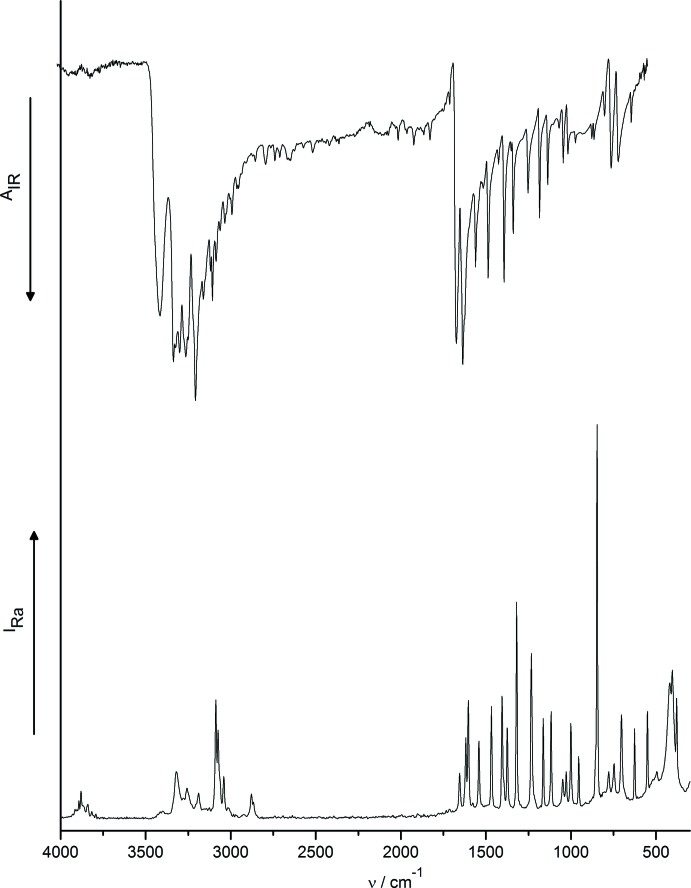
Shows the infrared spectrum (upper part) and Raman spectrum (lower part) of the title compound.

**Fig. 3.**
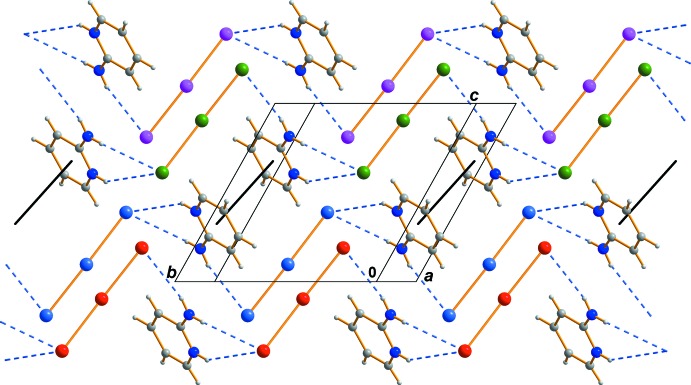
Pairwise twisted (green/violet and red/blue) hydrogen bonded chains run along [010].

**Fig. 4.**
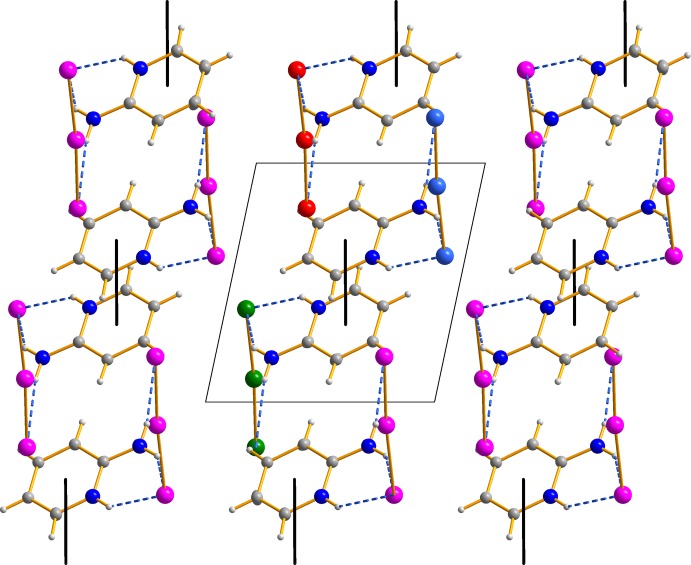
Showing the packing of rectangular rods constructed by pairwise twisted chains. π-π interactions are visualized by black lines connecting the centres of neighbouring rings.

## supplementary crystallographic information

### Comment

Aminopyridines are of general interest as they show biological activity (Bolliger *et al.*, 2011). Especially the monoprotonated cations are able to inactivate K^+^ channels reversibly (Muñoz-Caro & Niño, 2002). Another field of research related to 2-aminopyridinium salts is focused on their nonlinear optical properties (Srinivasan & Priolkar, 2013; Shkir, *et al.*, 2012; Periyasamy *et al.*, 2007). There are more than one hundred mono-protonated 2-aminopyridin-1-ium cations (*apy*H^+^) listed in the Cambridge Structural Database. Common to all is the protonation at the ring-nitrogen atom. Moreover, a short exocyclic C—N bond is typically for this cation which represents the so-called imino-form (Scheme 1). The electronic consequences of the mono-protonation of 2-Aminopyridine (Chai *et al.*, 2009; Testa & Wild, 1981) and the electronic structure of the resulting *apy*H^+^ monocation (Chapkanov, 2010) seem to be well understood. This contribution is part of our ongoing general interest in the hydrogen bonding of polyiodide salts (Reiss & Engel, 2002; Reiss & Engel, 2004; Meyer *et al.*, 2010). This applies in particular to the structural chemistry of aromatic nitrogen-containing polyiodide salts (Reiss & van Megen, 2012*a*).

The asymmetric unit of the title structure consists of one 2-aminopyridin-1-ium cation and one I_3_^-^ anion both located in general positions (Fig. 1). The geometric parameters of the *apy*H^+^ cation are in accord with the imino-form of a protonated 2-aminopyridine. The C–C and C–N bond lengths within the ring show C–N distances of 1.353 (5) and 1.354 (5) Å and C—C bond lengths ranging from 1.355 (5) to 1.411 (5) Å. The exocyclic C–N bond length is with 1.328 (5) Å very short, thus in the expected range for the imino-form of a protonated aminopyridine. Bond valence calculations for the *apy*H^+^ cation were performed using Brown's empirical method (Brown, 2009). The three different C–N bond lengths correspond to bond orders of 1.27 to 1.36, whereas the bond orders of the C–C bonds vary between 1.42 and 1.65 (Scheme 1). The geometric parameters of the triiodide anion are also in the typical range for a hydrogen bonded triiodide anion (*e.g.* van Megen & Reiss, 2012) with bond lengths of 2.8966 (3) and 2.9389 (3) Å and a bond angle of 176.02 (1)°. The Raman spectrum shows two intense signals at 126 and 115 cm^-1^ and a medium strong signal at 73 cm^-1^ which all are in excellent accord with the geometric parameters of the triiodide anion of the title structure and literature known examples (Deplano *et al.*, 1999). The Raman and the infrared spectrum show a vast number of bands from 4000 to 400 cm^-1^ which are in the expected ranges for the *apy*H^+^ monocation (Çırak, 2011; Fig. 2).

Cations and anions are connected by N–H ··· I hydrogen bonds. Each cation donates three un-bifurcated hydrogen bonds by the three hydrogen atoms attached to nitrogen atoms to two adjacent triiodide anions (Fig. 1). By these connections chains along the *b* direction are formed (Fig. 3). The hydrogen bonded chains are twisted pairwise to rectangular rods. These double chains (rods) (Fig. 3 and 4) are connected to adjacent ones by pyridine-pyridine interactions which are arranged in parallel with a plane to plane distance of 3.423 Å. This value is in excellent agreement with the results of *ab initio* calculations reported recently (Ninković *et al.*, 2012). In general, π-π interactions of pyridine moieties may play an important role in the biological system (Berl *et al.*, 2000) and are of significant interest in the structural chemistry of metal complexes with aromatic nitrogen-containing ligands (Janiak, 2000).

### Experimental

2-Aminopyridine (0.16 g; 1.7 mmol) was dissolved in 10 ml concentrated hydroiodic acid yielding a brown mixture. This mixture was heated to 90 °C and then slowly cooled to room temperature. Within 12 h needle-shaped, orange crystals grew from this solution. Elemental analysis (C_5_H_7_N_2_I_3_): calcd., %: C, 12.62; H, 1.48; N, 5.89; I, 80.01. Found, %: C, 12.07; H, 1.45; N, 5.60; I, 79.44. For details on the elemental analytical methods used, see: Reiss & van Megen (2012*b*); Egli (1969).

### Refinement

The coordinates of all hydrogen atoms were refined. The N-H distances were restrained to 0.85 (1) Å. It was possible to introduce individual U_iso_ values for the hydrogen atoms attached to nitrogen atoms, whereas for carbon bound hydrogen atoms U_iso_ values had to be set to 1.2U_eq_(C).

### Crystal data

**Table:**

C_5_H_7_N_2_^+^·I_3_^−^	*Z* = 2
*M**_r_* = 475.83	*F*(000) = 420
Triclinic, *P*1	*D*_x_ = 2.929 Mg m^−^^3^
*a* = 8.0446 (4) Å	Mo *K*α radiation, λ = 0.71073 Å
*b* = 8.9973 (5) Å	Cell parameters from 6254 reflections
*c* = 9.1464 (4) Å	θ = 3.1–32.6°
α = 117.805 (6)°	µ = 8.64 mm^−^^1^
β = 90.939 (4)°	*T* = 100 K
γ = 109.640 (5)°	Plate, orange
*V* = 539.46 (6) Å^3^	0.43 × 0.41 × 0.04 mm

### Data collection

**Table:**

Oxford Diffraction Xcalibur Eos diffractometer	2186 independent reflections
Radiation source: Sealed tube X-ray Source	2078 reflections with *I* > 2σ(*I*)
Equatorial mounted graphite monochromator	*R*_int_ = 0.021
Detector resolution: 16.2711 pixels mm^-1^	θ_max_ = 26.3°, θ_min_ = 3.1°
ω scans	*h* = −10→9
Absorption correction: analytical [*CrysAlis PRO* (Oxford Diffraction, 2009) based on expressions derived by Clark & Reid (1995)]	*k* = −11→11
*T*_min_ = 0.083, *T*_max_ = 0.698	*l* = −11→11
5668 measured reflections	

### Refinement

**Table:**

Refinement on *F*^2^	Hydrogen site location: difference Fourier map
Least-squares matrix: full	H atoms treated by a mixture of independent and constrained refinement
*R*[*F*^2^ > 2σ(*F*^2^)] = 0.018	*w* = 1/[σ^2^(*F*_o_^2^) + (0.015*P*)^2^ + 1.5*P*] where *P* = (*F*_o_^2^ + 2*F*_c_^2^)/3
*wR*(*F*^2^) = 0.041	(Δ/σ)_max_ = 0.001
*S* = 1.01	Δρ_max_ = 0.99 e Å^−^^3^
2186 reflections	Δρ_min_ = −0.59 e Å^−^^3^
117 parameters	Extinction correction: *SHELXL*, Fc^*^=kFc[1+0.001xFc^2^λ^3^/sin(2θ)]^-1/4^
2 restraints	Extinction coefficient: 0.0075 (2)

### Special details

**Table:**

Experimental. Analytical numeric absorption correction using a multifaceted crystal model based on expressions derived by Clark & Reid (1995).
Geometry. All e.s.d.'s (except the e.s.d. in the dihedral angle between two l.s. planes) are estimated using the full covariance matrix. The cell e.s.d.'s are taken into account individually in the estimation of e.s.d.'s in distances, angles and torsion angles; correlations between e.s.d.'s in cell parameters are only used when they are defined by crystal symmetry. An approximate (isotropic) treatment of cell e.s.d.'s is used for estimating e.s.d.'s involving l.s. planes.

### Fractional atomic coordinates and isotropic or equivalent isotropic displacement parameters (Å^2^)

**Table:**

	*x*	*y*	*z*	*U*_iso_*/*U*_eq_	
I1	0.09076 (3)	0.38499 (3)	0.61148 (3)	0.01704 (8)	
I2	0.19010 (3)	0.35305 (3)	0.90314 (3)	0.01422 (7)	
I3	0.26150 (3)	0.30976 (3)	1.18942 (3)	0.01955 (8)	
N1	0.2469 (4)	0.8865 (5)	0.8153 (4)	0.0250 (7)	
H11	0.162 (4)	0.782 (3)	0.774 (5)	0.031 (12)*	
H12	0.236 (6)	0.968 (5)	0.907 (3)	0.037 (13)*	
N2	0.4056 (4)	0.7899 (4)	0.6047 (4)	0.0182 (6)	
H2	0.325 (6)	0.686 (6)	0.561 (5)	0.026 (12)*	
C1	0.3907 (4)	0.9236 (5)	0.7495 (4)	0.0166 (7)	
C3	0.5493 (5)	0.8151 (5)	0.5310 (5)	0.0186 (7)	
H3	0.547 (5)	0.712 (6)	0.435 (5)	0.022*	
C4	0.6862 (5)	0.9812 (5)	0.5996 (5)	0.0217 (8)	
H4	0.783 (6)	0.990 (6)	0.549 (5)	0.026*	
C5	0.6744 (5)	1.1248 (5)	0.7472 (5)	0.0218 (8)	
H5	0.763 (6)	1.249 (6)	0.799 (5)	0.026 (11)*	
C6	0.5316 (5)	1.0977 (5)	0.8221 (5)	0.0192 (7)	
H6	0.520 (5)	1.191 (6)	0.915 (5)	0.023*	

### Atomic displacement parameters (Å^2^)

**Table:**

	*U*^11^	*U*^22^	*U*^33^	*U*^12^	*U*^13^	*U*^23^
I1	0.01701 (12)	0.01880 (13)	0.01929 (13)	0.00609 (9)	0.00315 (8)	0.01316 (10)
I2	0.01360 (12)	0.01388 (12)	0.01490 (12)	0.00556 (9)	0.00260 (8)	0.00688 (9)
I3	0.02394 (13)	0.02233 (13)	0.01393 (12)	0.01008 (10)	0.00330 (9)	0.00953 (10)
N1	0.0238 (17)	0.0189 (17)	0.0238 (17)	0.0040 (14)	0.0102 (14)	0.0070 (15)
N2	0.0174 (14)	0.0122 (15)	0.0205 (15)	0.0032 (12)	0.0019 (12)	0.0068 (13)
C1	0.0165 (16)	0.0179 (18)	0.0177 (17)	0.0060 (14)	0.0010 (13)	0.0111 (15)
C3	0.0182 (17)	0.0172 (18)	0.0216 (18)	0.0094 (14)	0.0050 (14)	0.0090 (15)
C4	0.0165 (17)	0.0216 (19)	0.028 (2)	0.0087 (15)	0.0073 (15)	0.0126 (17)
C5	0.0162 (17)	0.0159 (18)	0.028 (2)	0.0045 (15)	0.0002 (14)	0.0084 (16)
C6	0.0181 (17)	0.0158 (18)	0.0185 (18)	0.0063 (14)	−0.0013 (14)	0.0051 (15)

### Geometric parameters (Å, º)

**Table:**

I1—I2	2.9389 (3)	C1—C6	1.411 (5)
I2—I3	2.8966 (3)	C3—C4	1.355 (5)
N1—C1	1.328 (5)	C3—H3	0.93 (4)
N1—H11	0.849 (10)	C4—C5	1.400 (5)
N1—H12	0.847 (10)	C4—H4	0.91 (4)
N2—C1	1.353 (5)	C5—C6	1.358 (5)
N2—C3	1.354 (5)	C5—H5	0.97 (4)
N2—H2	0.83 (4)	C6—H6	0.91 (4)
			
I3—I2—I1	176.017 (9)	N2—C3—H3	115 (3)
C1—N1—H11	125 (3)	C4—C3—H3	124 (3)
C1—N1—H12	121 (3)	C3—C4—C5	118.3 (3)
H11—N1—H12	114 (4)	C3—C4—H4	117 (3)
C1—N2—C3	123.2 (3)	C5—C4—H4	124 (3)
C1—N2—H2	118 (3)	C6—C5—C4	120.9 (3)
C3—N2—H2	118 (3)	C6—C5—H5	116 (2)
N1—C1—N2	119.4 (3)	C4—C5—H5	123 (3)
N1—C1—C6	123.5 (3)	C5—C6—C1	120.0 (3)
N2—C1—C6	117.1 (3)	C5—C6—H6	121 (3)
N2—C3—C4	120.4 (3)	C1—C6—H6	118 (3)
			
C3—N2—C1—N1	−178.6 (3)	C3—C4—C5—C6	1.5 (6)
C3—N2—C1—C6	1.7 (5)	C4—C5—C6—C1	−1.2 (6)
C1—N2—C3—C4	−1.4 (5)	N1—C1—C6—C5	179.9 (4)
N2—C3—C4—C5	−0.3 (6)	N2—C1—C6—C5	−0.4 (5)

### Hydrogen-bond geometry (Å, º)

**Table:**

*D*—H···*A*	*D*—H	H···*A*	*D*···*A*	*D*—H···*A*
N1—H11···I1	0.85 (1)	2.99 (3)	3.698 (3)	142 (4)
N1—H12···I3^i^	0.85 (1)	2.89 (2)	3.709 (3)	164 (4)
N2—H2···I1	0.83 (4)	2.97 (5)	3.702 (3)	147 (4)

Symmetry code: (i) *x*, *y*+1, *z*.
